# Green Leaf Volatile Confers Management of Late Blight Disease: A Green Vaccination in Potato

**DOI:** 10.3390/jof7040312

**Published:** 2021-04-18

**Authors:** Neda Najdabbasi, Seyed Mahyar Mirmajlessi, Kevin Dewitte, Maarten Ameye, Marika Mänd, Kris Audenaert, Sofie Landschoot, Geert Haesaert

**Affiliations:** 1 Department of Plants and Crops, Valentin Vaerwyckweg 1, Faculty of Bioscience Engineering, Ghent University, 9000 Ghent, Belgium; seyedmahyar.mirmajlessi@ugent.be (S.M.M.); Kevin.Dewitte@ugent.be (K.D.); Maarten.Ameye@ugent.be (M.A.); Kris.Audenaert@ugent.be (K.A.); Sofie.Landschoot@ugent.be (S.L.); Geert.Haesaert@ugent.be (G.H.); 2 Institute of Agricultural and Environmental Sciences, Department of Plant Health, Estonian University of Life Sciences, Kreutzwaldi 5, 51014 Tartu, Estonia; marika.mand@emu.ee

**Keywords:** biological control, green leaf volatile, *Phytophthora infestans*, *Solanum tuberosum*, Z-3-hexenyl acetate

## Abstract

Yield losses of crops due to plant pathogens are a major threat in all agricultural systems. In view of environmental issues and legislative limitations for chemical crop protection products, the need to design new environmentally friendly disease management strategies has gained interest. Despite the unique capability of green leaf volatiles (GLVs) to suppress a broad spectrum of plant pathogens, their capacity to control the potato late-blight-causing agent *Phytophthora infestans* has not been well studied. This study addresses the potential role of the GLV Z-3-hexenyl acetate (Z-3-HAC) in decreasing the severity of late blight and the underlying gene-based evidence leading to this effect. Nine-week-old potato plants (*Solanum tuberosum* L.) were exposed to Z-3-HAC before they were inoculated with *P. infestans* genotypes at different time points. These pre-exposed potato plants exhibited slower disease development after infection with the highly pathogenic genotype of *P. infestans* (EU-13-A2) over time. Qualitative assessment showed that the exposed, infected plants possessed significantly lower sporulation intensity and disease severity compared to the control plants. Hypersensitive response (HR)-like symptoms were observed on the treated leaves when inoculated with different pathogen genotypes. No HR-like lesions were detected on the untreated leaves after infection. It was shown that the transcript levels of several defense-related genes, especially those that are involved in reactive oxygen species (ROS) production pathways were significantly expressed in plants at 48 and 72 h postexposure to the Z-3-HAC. The current work provides evidence on the role of Z-3-HAC in the increased protection of potato plants against late blight through plant immunity and offers new opportunities for the sustainable control of potato diseases.

## 1. Introduction

Plants are regularly attacked by a wide range of phytopathogens including viruses, bacteria, fungi, oomycetes and other microorganisms. The oomycete *Phytophthora infestans* (Mont.) de Bary, is one of the most destructive pathogens worldwide, responsible for the devastating late blight disease in potato and other solanaceous crops [1]. Without adequate control measures, this pathogen can cause major yield losses throughout the growing season, due to leaf destruction and tuber infections. Rapid outbreaks of this disease can be attributed to an increased rate of the asexual life stage, including sporangia and biflagellated zoospores [2]. Sporangia released into the atmosphere through rain splash or wind may cause new foliar infections. Under ambient temperature, the sporangia germinate directly by forming germ tubes or, indirectly, by producing motile flagellated zoospores that are able to swim towards stomatal pores on the leaf surface [3]. *P. infestans*, as a hemibiotrophic oomycete, exhibits an initial biotrophic phase with minimal symptoms on living host cells and a subsequent necrotrophic phase that can completely destroy a plant’s photosynthetic capacity within a short period of time [4]. In unprotected potato fields where disease incidence is high, the pathogen can lead to total crop failure within a period of 7–10 days [5]. Global average economic loss due to late blight amounts to about USD 6.7 billion annually, which is mainly due to high reduction in crop yields and extensive use of fungicides [6,7]. The costs of fungicide applications involved in disease management constitute a large part of expenses [8].

In the case of late blight control, most growers depend on repeated applications of a broad range of fungicides, such as: copper-based products, dithiocarbamates and cellulose synthesis inhibitors, etc. [9]. Despite their effectiveness against *P. infestans*, the side effects of these chemicals are a growing issue for both human health and the environment [10,11]. Increasing the frequency of chemical treatments along with the emergence of recombinant pathogen populations have led to problems associated with fungicide resistance in the field, e.g., phenylamids [5,12,13]. Oomycetes have different metabolic pathways, higher evolutionary potential and faster regeneration time compared to true fungi [14,15]. The growing public demand for reducing the use of conventional fungicides has promoted research to find innovative and environmentally friendly strategies to control *Phytophthora* species. However, yield stability remains the main challenge for farming systems [16]. Although the breeding of resistant plant varieties is the top priority, the development of biocontrol practices has also been proposed as an alternative sustainable strategy to manage *P. infestans* [13,17,18].

Among nonconventional approaches, secondary plant metabolites such as biogenic volatile organic compounds (BVOCs) are receiving attention as disease-control tools, particularly because of their low environmental impacts [19]. Green leaf volatiles (GLVs), one of the major BVOC groups, have also been reported to be inducers of defense-related responses in numerous plant species. Unstressed plant tissues continuously release GLVs in small amounts, whereas they rapidly release them in much greater amounts after stress [20], and they can also be emitted and induced systemically [21]. An increase in GLV emission can be caused by abiotic stimuli [22,23], herbivores [20,24,25] or plant pathogens [26,27,28]. GLVs play key roles in plant defense responses by exerting direct antimicrobial properties or by inducing priming. Priming allows the plant to prepare a quicker and more aggressive response to subsequent attacks by pathogens or herbivores [29,30,31]. These defense responses normally lead to a specific, localized form of programmed cell death (PCD) known as hypersensitive response (HR) [32]. Although the antimicrobial and antiherbivore activities of plant volatiles have been investigated by many researchers, scientific evidence on their impact on fungal, and particularly on oomycete, infections of plants is sparse.

GLV emission depends on the amount of damage caused by biotrophic or necrotrophic pathogens. Biotrophs establish a less aggressive association with the host’s cells compared to necrotrophs, which rapidly damage and kill host cells, resulting in enhanced GLV production [30]. Enhanced resistance of *Arabidopsis thaliana* to the necrotrophic pathogen *Botrytis cinerea* was demonstrated by treating leaves with the GLVs (E)-2-hexenal and (Z)-3-hexenal [33]. These C6-aldehydes conferred resistance in *Arabidopsis* by induced lignification, which acted as a barrier against penetration by the pathogen, and the accumulation of antifungal proteins *PDF1.2*, *PR-3* and the antifungal phytoalexin camalexin, which inhibits hyphal growth in plant cells. Similarly, the direct fungicidal activity of GLVs and the enhanced formation of camalexin in *Arabidopsis* seedlings with overexpressed hydroperoxide lyase (HPL) activity was responsible for improved defense responses against *B. cinerea* infection [34]. Treatment with hexanoic acid, a resistance priming compound structurally similar to GLVs, protected tomato plants from infection by the necrotrophic fungus *B. cinerea* by activating defense responses that prevent the harmful effects of reactive oxygen species produced by infection [35,36,37]. Transgenic tomato plants overexpressing *CsiHPL1*, a chloroplast-localized tea gene that encodes HPLs, can release wound-induced GLVs (Z)-hexenal and (Z)-3-hexen-1-ol and improve resistance to the necrotrophic fungus *Alternaria alternata* f. sp. *lycopersici* [38]. 

The biocidal activities of C6-aldehydes have been broadly discussed in the context of plant defense against various biotic stresses [34,39,40,41,42,43,44]. For example, pre-exposure to the C6-aldehyde Z-3-hexenyl acetate (Z-3-HAC) primed wheat seedlings for enhanced defense against the hemibiotrophic pathogen *Fusarium graminearum*, resulting in less necrotic symptoms on wheat ears compared to unexposed control plants. Moreover, this priming was accompanied by a large reduction in fungal growth in planta [45]. Information regarding other possible roles of GLVs in plant–fungal interactions has been comprehensively reviewed [20]. Recently, the possible role of the GLVs in plant defense against bacterial infections has also been uncovered [46]. Exogenous treatments of tomato plants with GLVs (Z)-3-hexenyl propionate and (Z)-3-hexenyl butyrate led to stomatal closure, *PR* gene induction and enhanced resistance to the bacterium *Pseudomonas syringae* pv. *tomato* [46]. Overall, the increased emission of GLVs such as E-2-hexenol, E-2-hexenal, Z-3-hexenol and Z-3-HAC by different plant species upon infection with pathogens represents an inducible defense mechanism against an impending attack that may affect neighboring plants as well as the plant itself. 

Despite the unique ability of GLVs to inhibit a wide range of fungal pathogens, their functions as defensive compounds against oomycetes remain poorly understood. The present work examines the hypothesis that treatment of potato plants with GLV Z-3-HAC prepares them for an enhanced defense against subsequent infection by *P. infestans*. Hence, the aims of this study were: (i) to investigate the protective potential of potato plants pre-exposed to Z-3-HAC against five genotypes of *P. infestans* in vivo, and (ii) to examine the possible defense responses of potato plants pre-exposed to Z-3-HAC against subsequent infection using molecular assays. 

## 2. Materials and Methods

### 2.1. Phytophthora Infestans Genotypes 

Different European genotypes of *P. infestans* including EU-1-A1, EU-6-A1, EU-13-A2, EU-36-A2 and EU-37-A2 were provided by the culture collection of Belchim Crop Protection, Belgium. To ensure aggressiveness had not been lost, they were regularly transferred to potato slices (cv. Bintje) for host passage, as described in an earlier study by Najdabbasi et al. [18]. Cultures of *P. infestans* were grown on rye-B agar (containing 0.005% b-sitosterol) (Merck, Darmstadt, Germany) at 18 °C in the dark for 12–14 days before use. For inoculum preparation, sporulating plates were gently washed with sterile distilled water (SDW), and sporangia concentration was determined using a hemocytometer (average of three readings) and adjusted to a final concentration of 5 × 10^4^ sporangia mL^−1^. Zoospore release was then stimulated by cold temperatures at 4 °C for 2 h and their motility was checked under the microscope before inoculation.

### 2.2. Plant Material

Disease-free potatoes (cv. Bintje) were raised through micropropagation and used as plant material. To improve germination, potato tubers were washed thoroughly in tap water and soaked in a 0.3% gibberellic acid-3 (Merck, Darmstadt, Germany) solution for 5 min. Then they were placed in paper bags and kept in constant darkness at 22 °C. Fifteen-day-old sprouts were excised from tubers and used as explants for shoot regeneration. Nodal explants, each containing an axillary bud and cut into 5 mm slices, were rinsed with tap water and then 70% ethanol for a few seconds. They were treated with 10% Chlorex (5.25% NaOCl, Colruyt, Halle, Belgium) and one droplet of 0.1% Tween 80 (Sigma-Aldrich, St. Louis, MO, USA) for 15 min and then rinsed with SDW three times. The explants were cultured in MS medium [47] containing macro- and micronutrients and vitamins (Duchefa Biochemie, Haarlem, The Netherlands) (nicotinic acid: 0.4 mg/L, myo-inositol: 100 mg/L, glycine: 2 mg/L, thiamine hydrochloride: 0.1 mg/L, pyridoxine hydrochloride: 0.5 mg/L), supplemented with sucrose (30 g/L) and plant agar (7 g/L). All cultures were incubated at 25 °C under cool white fluorescent lights that provided a 16:8 h photoperiod. After 5 weeks, rooted plantlets were transplanted from the medium and acclimatized in pots (4 L) containing standard potting soil (complex NPK fertilizers 14-16-18, pH 5–6.5, organic and dry matter: 25% and 20%, respectively) for their establishment. The potato seedlings were grown at 22 °C, 60% RH and a 16:8 h photoperiod in a plant growth chamber.

### 2.3. Application of Z-3-HAC to Potato Plants

**Figure 1:**
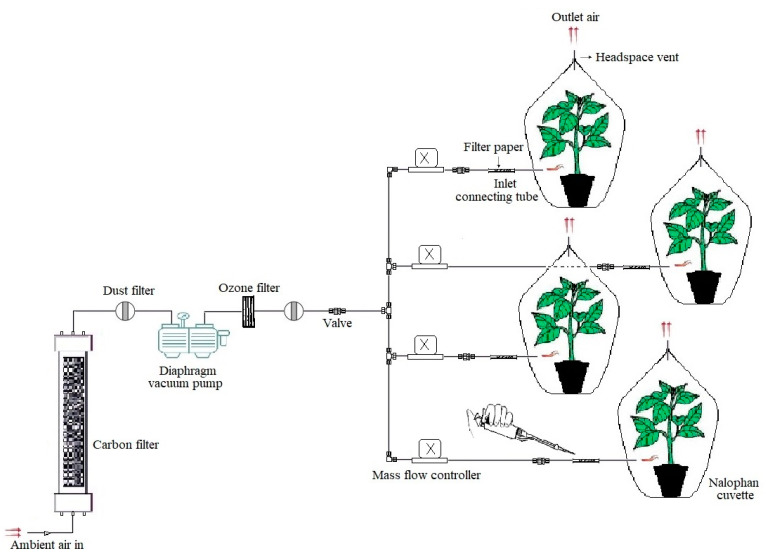
Schematic illustration of the modified push–pull cuvette system used in this study. Z-3-HAC (55 µL) was applied on a piece of filter paper inside the inlet-connecting tubes over 6 h with 1 h intervals. Tubing and connections were made out of perfluoroalkoxy alkane or stainless steel (Swagelok, Solon, OH, USA).

To evaluate whether GLV treatment improved defense responses, potato plants were treated with Z-3-HAC using a modified dynamic push–pull cuvette system, as described by Ameye et al. [44]. The volatile compound used in this study was acquired as a commercial preparation with a nominal purity of least 97% (Sigma-Aldrich, St. Louis, MO, USA). Each potato plant was placed in a nalophan cuvette (NA 300, Foodpack, Harderwijk, The Netherlands) in order to be exposed to the GLV Z-3-HAC prior to infection with *P. infestans*. Nine-week-old potato plants were separately exposed to 330 µL of Z-3-HAC (9.13 µM) through filter paper positioned inside the inlet-connecting tubes (Swagelok, Solon, OH, USA). To prevent exposing plants to phytotoxic levels of the compound, 55 µL of Z-3-HAC was consecutively applied over 6 h with 1 h intervals. Ambient air was constantly pushed into the nalophan cuvettes at a rate of 600 mL min-1 through a diaphragm vacuum pump (N035AN.18; KNF Neuberger GmbH, Freiburg i. Br., Germany) that was installed after a dust filter (2 mm pore size Zefluor PTFE membrane filter, Pall, MI, USA). The flow rate of the air stream was regulated by mass flow controllers (GF40; Brooks Instruments, Hatfield, PA, USA), allowing a uniform airflow over the plant. To eliminate Z-3-HAC reacting with contaminants or pollutants (e.g., ozone) found in the incoming air, an active carbon filter (Airpel 10; Desotec, Roeselare, Belgium) was placed inside of a stainless steel canister and also an ozone filter (ETO342FC002A; Ansyco, Karlsruhe, Germany) was installed (Figure 1). The residual airflow was exhausted from the nalophan cuvette through a headspace vent, removing trace amounts of the GLV compound. To avoid recirculation of airflow, the inlet tube of the carbon filter was located outside the growth chamber. Subsequently, the treated plants were inoculated with sporangial suspensions of *P. infestans* genotypes according to experiments that are described below. Untreated plants were considered to be the negative control across all experiments. Potato leaves were visually checked for phytotoxicity symptoms over time.

### 2.4. Biocidal Activity of Z-3-HAC In Vivo 

To examine the potential impact of the pre-exposure of potato plants to Z-3-HAC on subsequent infection with *P. infestans*, detached leaflet bioassays were conducted at two different time points (1 and 24 h after exposure), according to Najdabbasi et al. [18]. Briefly, three composed leaves, equivalent to fifteen leaflets per treatment, pre-exposed to the Z-3-HAC, were cut and set abaxial surface down on foam inside the plastic trays (60 × 50 × 20 cm) containing distilled water. The inoculation of leaflets was performed using a single droplet (20 μL) of sporangial suspension at the center of each leaflet. Detached leaflets inoculated with SDW were included in all bioassays as a negative control for each treatment. Trays were then wrapped in transparent polythene bags to produce high relative humidity and kept in a growth chamber at 18 °C with a 16 h photoperiod. Disease assessments were performed 7 days after inoculation at each time point. Disease symptoms were calculated on an arbitrary 1–3 grading scale for each treatment, where 1: presence of necrotic/non-sporulated lesion, 2: presence of sporulated lesion, 3: hypersensitive response (HR)-like lesion. Likewise, sporulation intensity (SI) was classified by stereomicroscope using a semiquantitative grading scale ranging from 0–3, where: 0: no sporulation, 1: weak sporulation, 2: medium sporulation, 3: high sporulation. A disease severity index (DSI) on a percentage basis was additionally assessed by measuring lesion size using a 1–9 grading scale [48], where 0: no infection, 1: small lesion on the inoculation point with the area < 10% of the whole leaflet, 3: lesion area < 20%, 5: lesion area < 30%, 7: lesion area between 30–60%, and 9: lesion area > 60%, according to the following equation: 

DSI (%) = [Σ (class occurrence × grading class score)/(total leaflets in a set) × (maximum grading)] × 100. Each experiment was repeated twice with three replicates per treatment.

### 2.5. Visualization of Cell Death 

To determine whether the pre-exposure of potato plants to the GLV Z-3-HAC resulted in the formation of cell-death lesions upon infection with *P. infestans*, the staining of dead cells was carried out using Evans blue dye (Sigma-Aldrich, St. Louis, MO, USA). The inoculated leaves were cut and stained with 0.5% (*w*/*v*) Evans blue solution dissolved in distilled water, as described by Wang et al. [49] with some modifications. The whole leaflets were put on a small piece of gauze pad and submerged in the dye. Leaflets were harvested 1 h after incubation, washed in distilled water and immediately examined under the microscope. Whole cells that were entirely stained dark blue were counted as dead cells.

### 2.6. RNA Extraction and cDNA Synthesis

Potato leaves were directly frozen in liquid nitrogen and kept at −80 °C until further processing. About 0.5 g of leaf samples were ground to powder in liquid nitrogen with a mortar and pestle, and total RNA was extracted from 100 mg of ground tissue from both treated and untreated plants using TriReagent (Sigma-Aldrich, St. Louis, MO, USA), according to the manufacturer’s specification, and quantified using a Quantus fluorometer (Promega, Leiden, The Netherlands). Four leaflets were randomly taken from the pre-exposed potato plants and pooled for each time point (1, 12, 24, 48 and 72 h after exposure), according to the respective treatments: unexposed, noninfected plants as control, exposed plants, exposed plants with subsequent infection with sporangial suspension of *P. infestans* and unexposed plants inoculated with sporangial suspension of *P. infestans*. Normally, 2 µg of total RNA were reverse-transcribed to the first-strand complementary DNA (cDNA) using the GoScript Reverse Transcription System (Promega Corporation, Madison, WI, USA) according to the manufacturer’s instruction. The presence of cDNA was confirmed by agarose gel electrophoresis. cDNA was stored at −20 °C if not used immediately.

### 2.7. Gene Expression Analysis

The effect of the GLV on defense-related genes’ expression against *P. infestans* infection on 9-week-old potato plants pre-exposed to Z-3-HAC was investigated using quantitative reverse transcription PCR (RT-qPCR) at the five time points as mentioned above. For each time point, three biological replicates and two technical replicates were performed. RT-qPCR was performed using a CFX96 Touch Real-Time PCR Detection System (BIO-RAD, Temse, Belgium). Each reaction was carried out with 2 µL of cDNA template, 6.25 μL of GoTaq qPCR Master Mix (Promega, Leiden, The Netherlands), 0.62 μL of each primer (5 μM), 0.20 μL of CXR reference dye (Promega, Leiden, The Netherlands) and nuclease-free water up to a total volume of 12 μL. The thermal cycling conditions consisted of an initial denaturation at 95 °C for 5 min followed by 55 cycles of 95 °C for 10 s, 53 °C for 20 s and 72 °C for 30 s. A melting curve temperature profile was attained by heating to 95 °C, cooling to 65 °C and gradually heating to 95 °C at a rate of 0.5 °C per 10 s. The specificity of each primer pair was confirmed if reactions gave a single peak in melt-curve analyses. For normalization of the data, two reference genes, *β-tubulin* and *actin*, were used. The ΔΔCt method (2^−ΔΔCt^) was employed to quantify the relative gene expression levels between different treatments using the Ct values generated by the RT-qPCR system [50]. The primers used are presented in Table 1.

### 2.8. Statistical Analysis

All the descriptive and statistical analysis between different treatments was performed using “R” software (version 2.15.3). The data are displayed as barplots describing disease severity variations and expression profiles of genes. Since the homoscedasticity assumption (Levene’s test) and normality assumption (Shapiro–Wilk test) for an ANOVA were not satisfied, a nonparametric Kruskal–Wallis test was performed to determine overall differences followed by a post hoc Dunn’s test used for multiple comparisons (*p*-value < 0.05). Chi-squared tests were also used to test for the relative proportions between exposed leaves and unexposed control leaves. The amplification efficiency representative for each gene was also determined using the Representational State Transfer (REST) software [54].

## 3. Results

### 3.1. Effect of Pre-Exposure Z-3-HAC on Late Blight Symptoms 

**Figure 2:**
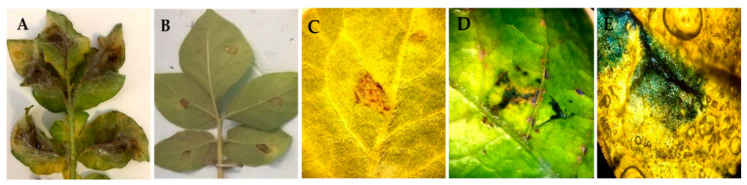
Development of hypersensitive response (HR)-like lesions on detached potato leaves pre-exposed to Z-3-HAC after spot-inoculation with *P. infestans* sporangial suspension (5 × 10^4^ sporangia mL^−1^) at 1 h postexposure. (**A**) unexposed, infected leaf; (**B**,**C**) restricted area consisting of HR-like lesions; (**D**,**E**) Evans-blue-based detection of dead cells in lesions. Dead cells in HR-like lesions demonstrated by Evans blue stained in blue. Symptoms were assessed seven days after infection.

The experiment was set up with nine-week-old healthy potato plants, originating from in vitro culture, that were pre-exposed to the Z-3-HAC. Disease symptoms were determined after infection by five genotypes of *P. infestans* according to lesion characteristics on potato leaves at 1 and 24 h postexposure. A wide range of symptoms caused by different *P. infestans* genotypes were observed on leaves seven days after inoculation: from sporulated lesions to necrotic lesions resembling HR. The most effective defense response was against genotypes EU-36-A2 and EU-1-A1 with HR-like symptoms on all inoculated leaflets at 1 h postexposure; no sporulated lesion was observed. These symptoms appeared as areas of dead cells at the inoculation point 2–3 days after infection that increased in size without any additional damage on the leaf surface while the infection progressed. A strong positive reaction (dark blue) to Evans blue staining was observed in HR-like lesions (Figure 2). 

**Figure 3:**
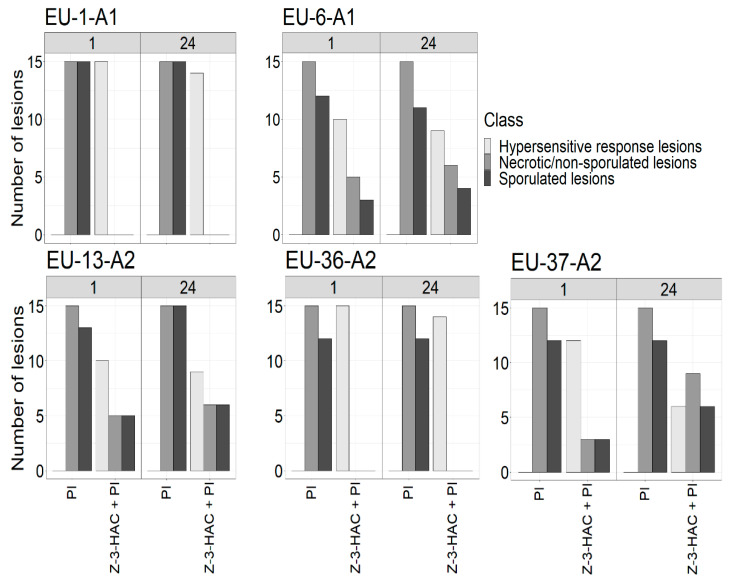
Number of lesions belonging to the different symptom classes (hypersensitive response, necrotic/non-sporulated lesions and sporulated lesions) on unexposed potato leaves inoculated with various *P. infestans* genotypes (EU-1-A1, EU-6-A1, EU-13-A2, EU-36-A2, EU-37-A2) (PI) and on potato leaves pre-exposed to the Z-3-HAC and subsequent infection with *P. infestans* genotypes (Z-3-HAC + PI) at 1 and 24 h postexposure. The class distribution between treated and untreated leaves was significantly different according to the chi-square test (*p*-value < 0.01). All measurements were assessed seven days after infection.

Comparatively, leaves inoculated with EU-6-A1, EU-13-A2 and EU-37-A2 developed substantial sporulated lesions at the first time point (Figure 3). Although the number of HR-like lesions remained high on leaves after inoculation with the different genotypes at 1 h postexposure, this number slightly decreased at 24 h post-exposure on all leaves. Moreover, the inhibition of sporulation on leaves infected at 1 h postexposure was higher than those infected at 24 h postexposure, from which EU-13-A2 showed the highest number of sporulated lesions of any time point. No HR-like lesions were detected on unexposed control leaves after infection with the genotypes of *P. infestans*.

### 3.2. Effect of Pre-Exposure to the Z-3-HAC on Intensity of Sporulation 

**Figure 4:**
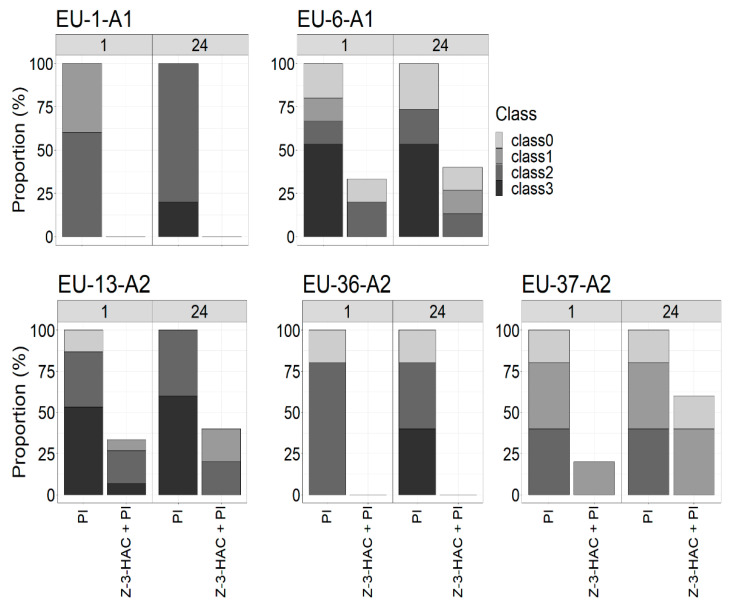
Sporulation intensity proportion of potato leaves belonging to the four sporulation levels (0 = no sporulation, 1 = weak sporulation, 2 = medium sporulation, 3 = high sporulation) on unexposed leaves inoculated with various *P. infestans* genotypes (EU-1-A1, EU-6-A1, EU-13-A2, EU-36-A2, EU-37-A2) (PI) and on leaves pre-exposed to the Z-3-HAC and subsequent infection with *P. infestans* genotypes (Z-3-HAC + PI) at 1 and 24 h postexposure. The class distribution between treated and untreated leaves was significantly different according to chi-square test (*p*-value < 0.01). All measurements were assessed seven days after infection.

To verify whether Z-3-HAC pre-exposure to potato plants influences sporangia production in different *P. infestans* genotypes, sporulation intensity was assessed on leaves inoculated at 1 and 24 h postexposure. Seven days after inoculation, signs of mycelial growth were observed on almost all leaflets. However, sporulation intensity differed amongst genotypes, ranging from no sporulation to high sporulation with overly branched mycelia (Figure 4). Overall, *P. infestans* genotypes expressed a lower sporulation intensity on leaflets inoculated at 1 h postexposure rather than at 24 h. The sporulation intensity of EU-13-A2 was higher on treated and untreated leaflets compared to other genotypes and had the highest proportion of leaflets showing class 3 sporulation intensity. While EU-37-A2 showed sporulation on a higher proportion of treated leaflets 24 h postexposure, sporulation intensity was never higher than class 1 for this genotype for that treatment. In comparison the genotypes EU-6-A1 and EU-13-A2 showed higher classes of sporulation intensity than EU-37-A2 on treated leaflets at 24 h postexposure. Due to the presence of HR-like lesions on pre-exposed leaflets after inoculation with EU-36-A2 and EU-1-A1 genotypes, no sporulated lesions were observed at any time point.

### 3.3. Effect of Z-3-HAC Pre-Exposure on the Severity of Late Blight Disease

**Figure 5:**
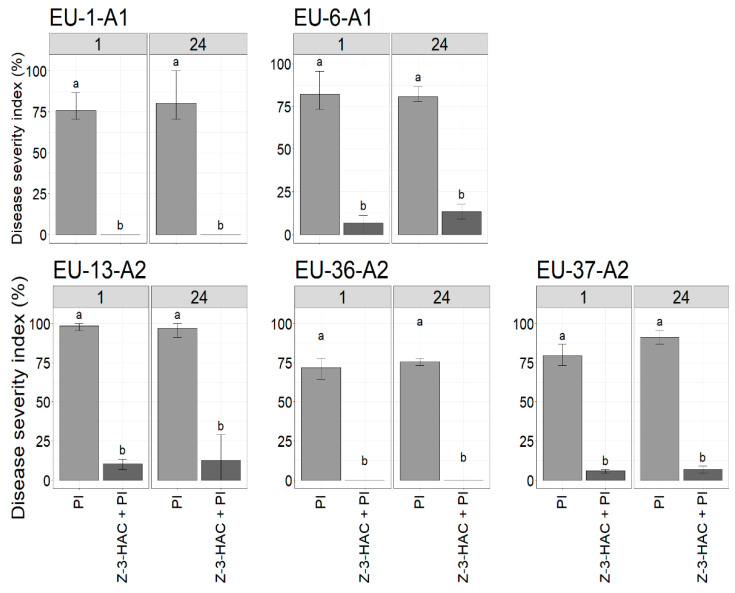
Barplots showing the variation in the late blight disease severity index (%) between unexposed potato leaves inoculated with various *P. infestans* genotypes (EU-1-A1, EU-6-A1, EU-13-A2, EU-36-A2, EU-37-A2) (PI) and leaves pre-exposed to the Z-3-HAC and subsequent infection with *P. infestans* genotypes (Z-3-HAC + PI) at 1 and 24 h postexposure. Bars with different letters represent values that are significantly different according to Dunn’s test (*p*-value > 0.05). All measurements were assessed seven days after infection.

A disease severity index (DSI) based on the size of disease lesions was used to evaluate Z-3-HAC on late blight development in pre-exposed plants. Seven days after infection, a wide range of results between *P. infestans* genotypes was observed at different time points, ranging from complete control to severe infection (Figure 5). As shown in Figure 5, late blight was completely suppressed in treated plants when inoculated with EU-1-A1 and EU-36-A2 at all time points. Leaves of potatoes pre-exposed to the Z-3-HAC resulted in a significantly lower disease severity score compared to the untreated control plants (*p*-value < 0.05). When comparing the same genotype, no significant differences in DSI were found between the different time points (1 and 24 h postexposure) for treated plants. Additionally, different levels of disease severity were observed between different genotypes on untreated plants, with genotype EU-13-A2 exhibiting the highest DSI value of ~ 98%. These findings strengthen the conclusion that EU-13-A2 was the most virulent genotypes amongst those tested and so was selected for subsequent experiments to assess distinct changes in defense-related gene expression in potato plants pre-exposed to the Z-3-HAC over time. 

### 3.4. Expression Analysis of Z-3-HAC-Regulated Genes in Potato Plant 

**Figure 6:**
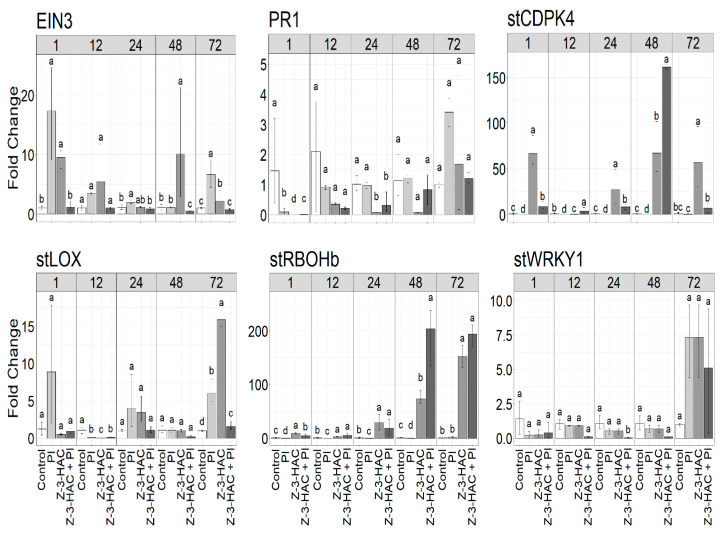
Expression profiles (fold change) of *stPR-1, stLOX, stWRKY1, EIN3, stRBOHb* and *stCDPK4* genes in potato plants 1, 12, 24, 48 and 72 h after exposure with the GLV Z-3-HAC. Pre-exposed plants were challenged with sporangial suspension of *P. infestans* (EU-13-A2) after exposure. PI: not pre-exposed, infected; Z-3-HAC: pre-exposed, not infected; Z-3-HAC + PI: pre-exposed, infected; control: not pre-exposed, not infected. Bars sharing the same letters represent values that are not significantly different according to Dunn’s test (*p* > 0.05).

Since pre-exposure of potato plants with the GLV Z-3-HAC reduced qualitative parameters including disease symptoms and sporulation capacity, an induced plant defense response was hypothesized. For the quantitative assessment of plant responses, the expression of several genes involved in plant defense was monitored in pre-exposed potato plants, originating from in vitro culture, during subsequent infection with *P. infestans* (EU-13-A2) using RT-qPCR at five time points (1, 12, 24, 48 and 72 h postexposure). The transcript levels of *stPR-1*, *stLOX*, *stWRKY1*, *EIN3*, *stRBOHb* and *stCDPK4* genes were upregulated in potato plants pre-exposed to the GLV Z-3-HAC (Figure 6). The highest induction of the expression of genes was detected at 48 and 72 h postexposure, with *stRBOHb* showing the highest level of expression at 48 h postexposure (up to ~ 152-fold, *p*-value < 0.05). Moreover, subsequent infection significantly increased the expression levels of *stRBOHb* and *stCDPK4* at 48 h postexposure, up to ~ 203-fold and ~ 161-fold, respectively (*p*-value < 0.05). Indeed, the suppression of *stRBOH* and *stCDPK* genes by *Phytophthora* infection was observed at different time points, where it was negated by Z-3-HAC exposure. For *stLOX, stWRKY1* and *EIN3*, a higher level of expression was observed in the exposed, noninfected plants in comparison with the exposed, infected plants at 24, 48 and 72 h postexposure. These genes were slightly induced by the Z-3-HAC at different time points after the challenge inoculation with *P. infestans*. A large upregulation of *EIN3* and *stLOX* genes in unexposed plants at 1 h after inoculation was also observed. Interestingly, the transcript level of the *stPR-1* gene showed higher expression in the unexposed, infected plants than the exposed plants at any time point. Overall, the minimum proportion of expression in most differentially expressed genes was found at 12 and 24 h postexposure. 

## 4. Discussion

Seeking nature-based solutions has been a long-standing strategy in disease control management, and natural-product-derived compounds are established to be the most effective for maintaining the balance between development and environmental protection. Volatile-mediated induced resistance has been gaining increased attention in crop production as the major mechanism mediating the protection of plants. C6-aldehyde compounds, which are known as green leaf volatiles (GLVs), have been reported to negatively affect pathogen performance on host plants. Although the elicitation of GLV-mediated induced resistances have been shown for several crops [37,45,46,55,56,57,58,59], to our knowledge, this study was the first attempt to investigate whether GLVs can be used to promote natural plant resistance of the potato plant to *Phytophthora* infection. Indeed, no previous research has studied GLV-induced resistance in the potato plant, despite the potato being one of the world’s most important crops.

In the first part of this study, the antioomycidal capacity of the Z-3-HAC compound against five pathogenic genotypes of *P. infestans* was assessed based on qualitative analyses using detached leaflet bioassays. We demonstrated the impact of Z-3-HAC exposure to inhibit late blight in planta. On the leaves treated with Z-3-HAC, *Phytophthora* infection was significantly lessened compared to Z-3-HAC untreated plants. Z-3-HAC exposure resulted in significantly better control against *P. infestans* genotypes in comparison to untreated control plants; although the effect was less pronounced on leaves inoculated at 24 h compared to 1 h postexposure. The GLV treatment resulted in necrotic lesions, resembling HR, at the site of inoculation on the leaf surface with no visible phytotoxic effects when inoculated at any time point. The Z-3-HAC-induced HR in potato leaflets manifested as distinguishable microlesions that developed into confined macro HR lesions at the site of inoculation. Necrotic lesions containing dead cells were clearly distinguished by dark blue staining with Evans blue. Due to the semipermeable property of the cells, living cells exclude the dye; dead cells are incapable of rejecting the dye and are thereby stained blue [60]. However, the number of HR-like lesions in all pre-exposed leaves slightly varied at different time points. HR is a potent defense response against pathogen attack, which leads to cell-death induction at the site of pathogen penetration [61]; this limits infection to a specific area of the plant tissue. HRs induced by different types of pathogens, like bacteria, fungi, oomycetes and viruses, influence interactions with the host plant. HR-associated cell death is normally linked to the involvement of specific genes in host plants with biotrophic pathogens and is often not effective against necrotrophic pathogens as they kill host cells to feed on and complete their life cycle [62]. Unlike biotrophic and necrotrophic fungi, hemibiotrophic oomycete *P. infestans* has developed a more complex interaction with its host. This begins with an asymptomatic phase of infection, which is truly biotrophic and crucial for disease establishment, and subsequently switches to a visible necrotrophic phase wherein cell death is frequently associated with exponential growth and extensive sporulation, initiating a new infection cycle [63]. In our experiment, no HR lesions were phenotypically observed on untreated, inoculated leaves even 10 days after inoculation, at which point they were entirely destroyed by the infection (data not shown). The onset of disease began with small necrotic lesions on the control leaves that quickly expanded throughout the leaves, representing the necrotrophic phase of *P. infestans*. In contrast, pre-exposed leaves with HR-like lesions never showed any significant damage on the leaf as the infection progressed. 

Based on our results, exposure of potato plants to Z-3-HAC for 7 h reduced the severity of late blight. It was shown that Z-3-HAC pre-exposure in potato plants decreased DSI, as well as the sporulation intensity of *P. infestans* genotypes, resulting in increased protection of leaves compared to the untreated control. Additionally, treatment with Z-3-HAC decreased late blight development for the whole plant over time (data not shown). It has been shown that plant cuticles can passively adsorb volatile compounds that are highly persistent on the surface of plant foliage, thus applying inhibitory effects against phytopathogens and providing associational resistance [64,65]. The effect of Z-3-HAC pre-exposure on biocontrol efficacy is in accordance with the findings of Ameye et al. [45], who showed that wheat seedlings pre-exposed to the GLV Z-3-HAC exhibited significantly smaller necrotic lesions and a lower infection rate when inoculated with a hemibiotrophic pathogen, *Fusarium graminearum*, in comparison with the unexposed treatments. In our experiments, all genotypes of *P. infestans* were pathogenic on the unexposed potato leaves with consistently high or low level of aggressiveness. Among the *P. infestans* genotypes tested in this study, EU-13-A2 was the most pathogenic genotype, as it showed higher sporulation intensity and DSI than other genotypes. This agrees with the results of Najdabbasi et al. [18], who concluded that EU-13-A2 was the most aggressive genotype in terms of aggressiveness and sporulation intensity on both potato tubers and leaves. The European EU-13-A2 has been introduced as a dominant population of *P. infestans* in Europe since 2004, where genotypes EU-1-A1 and EU-6-A1 had continuously existed in the population for over a decade [66,67,68]. Nevertheless, two genotypes, EU-36-A2 and EU-37-A2, have lately emerged in different parts of Europe, according to the EuroBlight monitoring network [69]. Genome analyses of the genotype EU-13-A2 revealed high levels of individual polymorphism, amino acid replacement and variation in gene expression levels resulting in diverse aggressive behavior during infection and the capacity to cause disease on formerly *P. infestans*-resistant potato cultivars [66]. However, contradictory findings have been reported in the literature for aggressiveness and sporulation intensity within lineages on different hosts [69,70,71]. For example, Dey et al. [72] found some variations in pathogenicity within the EU-13-A2 clonal lineages, in which the pathogen showed greater aggressiveness on potato than on tomato plants. They also identified multilocus genotypes (MLGs) from tomato plants that did not infect potato plants and vice versa. These inconsistencies fortify the theory that mutations in various pathogen genotypes may cause high aggressiveness towards a particular host, which may have evolved independently in different geographic locations [73]. This was also in agreement with earlier studies that revealed A2 genotypes to be more aggressive than A1 genotypes, classified according to clonal lineage [66,67,68,74]. 

Since late blight symptoms were limited to susceptible potato leaves pre-exposed to the GLV Z-3-HAC, this compound might contribute to the activation of the systemic defense mechanisms in the plant, which may eventually result in subsequent priming. Upregulation of defense-related genes following stronger defense responses in the treated plants with natural volatile compounds is shown by priming [30,31]. To examine the potential of Z-3-HAC to activate plant immunity, the expression patterns of defense-related genes were analyzed in potato leaflets pre-exposed to the GLV, followed by infection with *P. infestans*. Genes encoding respiratory burst oxidase homolog B (*stRBOHb*), transcription factor 1 (*stWRKY1*), lipoxygenase (*stLOX*), pathogenesis-related protein 1 precursor (*stPR1*), calcium-dependent protein kinase 4 (*stCDPK4*) and ethylene insensitive 3 (*EIN3*) were selected (*st*; *Solanum tuberosum*) as defense markers. In the present study, gene expression analysis indicated that pre-exposure of potato plants to Z-3-HAC enhanced expressions of *stLOX* and *stWRKY1*, whereas these genes were partially suppressed after infection. The expression of *stLOX* was associated with the upregulation of *stWRKY1* transcription factor at 48 h postexposure. Z-3-HAC pre-treatment could considerably induce the expression of *stRBOHb* and *stCDPK4* genes at 48 h postexposure and significantly boost the expression of these genes in response to subsequent infection by *P. infestans*. Genetic studies indicate that both *RBOH* and *CDPK* are key regulators of reactive oxygen species (ROS) production and exhibit extensive pleiotropy in plants [75,76,77,78,79,80]. Therefore, this is the strongest clue that ROS production confers protection to Z-3-HAC-induced priming, as *P. infestans* alone suppressed that response. The expression of active *stCDPK* induces ROS production and HR-like cell death through the direct N-terminal phosphorylation of NADPH oxidase RBOHD on potato leaves at the infection site of *P. infestans* [78,79]. In this context, the accumulation of ROS at the cell surface has been involved in plant defense responses against secondary infections by strengthening host cell walls via the crosslinking of glycoproteins [81,82]. The production of ROS during the oxidative burst reaction is a hallmark of defense responses in plant cells undergoing HR [61,83,84,85].

In contrast to the *stLOX, stWRKY1, stRBOHb* and *stCDPK4* marker genes, *stPR-1* was less expressed in plants after exposure. Indeed, a minor induction of gene expression after treatment with the Z-3-HAC was found, so that no significant change in the transcript levels of *stPR-1,* compared with the infected treatment, was observed in plant leaves, except at 1 h postexposure. This is in accordance with the study of Bate and Rothstein [86], who exposed *Arabidopsis* seedlings to the GLV E-2-HAL, where it induced *PAL* (phenylalanine ammonia-lyase)*, LOX* and *AOS* (allene oxide synthase) genes known to be involved in the plant’s defense response, whereas genes encoding *PR-1* and *PR-2* were not induced. *PR* genes may be activated in the presence of pathogen-derived cues and may completely or partially suppress pathogen attack [87,88,89]. These findings contradict the studies of Schröder et al. [90] and Hoegen et al. [91] who demonstrated that the expression of *PR-1* and *PR-2* genes were systemically upregulated in potato plants after infection by *P. infestans*, suggesting the induction of the SA-signaling pathway in response to the recognition of the pathogen by potato plants. The function of PRs is not fully understood [92] and hence, the reduced expression of *PR-1* and *PR-2* genes described in this study in the presence of *P. infestans* cannot be irrefutably linked to a mechanism that confers resistance to late blight in potato plants. However, there is a hypothesis that *PR-1* may be involved in plant PCD during HR through a protease cascade [93]. For transcription factor (TF) *EIN3*, no increased gene expression was observed after inoculation of pre-exposed potato plants with *P. infestans* (Z-3-HAC + PI) at any time point, but the expression slightly increased in untreated and treated leaves. Increasing the level of *EIN3* expression in potato plants pre-exposed to the Z-3-HAC might improve defense. There is also evidence for the implication of the *EIN3* family of TFs gene in defense responses to pathogen attack and environmental stimuli [94,95,96]. *EIN3* is a key regulator of the ethylene (ET) signaling pathway involved in the regulation of HR-like cell death and defense responses [97,98].

Since *P. infestans* switches to a destructive necrotrophic phase during infection, the increased expression of genes in treated plants at 72 h postexposure might have contributed to inducible defense systems. For example, the *stRBOHb* gene gradually started increasing in expression in leaves when exposed to Z-3-HAC 24 h before infection so that it was largely upregulated during later stages of its biotrophic phase. This was associated with the involvement of ROS, causing a higher number of necrotic lesions on leaves. Furthermore, there was evidence for an SA-controlled signaling pathway in the regulation of gene expression due to *P. infestans* infection. According to gene expression profiles, the enhanced coexpression of genes involved in SA- or JA-signaling pathways was observed in plants pre-exposed to Z-3-HAC. However, the transcript levels of *stWRKY1*, *stRBOHb* and *stCDPK4* genes were larger compared to those on the JA-dependent gene *stLOX* in GLV-treated plants after the pathogen challenge. We found strong expression of *stRBOHb* and *stCDPK4* genes to be upstream of SA, upon infection with the pathogen at 48 h postexposure. Nevertheless, a high level of *stLOX* expression was found in treated leaves at 72 h postexposure. Due to the hemibiotrophic lifestyle of *P. infestans*, it can be postulated that both SA and JA could play a pivotal role in regulating plant defense response against this pathogen. Previous studies have reported this biphasic SA- and JA-mediated basal defense response against *P. infestans* in potato plants [99,100]. A recent study indicated that intact SA-signaling is essential for potato defenses against the necrotrophic pathogen *Alternaria solani* [101]. This contrasts with the documented minimal impact of SA-dependent defense responses of the model plant *A. thaliana* on necrotrophic pathogens that are essential for resistance to biotrophic and hemibiotrophic pathogens [102]. Furthermore, Scala et al. [20] showed that the growth of the biotrophic bacterium *Pseudomonas syringae* was significantly reduced in *A. thaliana* plants that showed reduced production of the GLV E-2-HAL. This also correlated with lower JA levels and higher SA levels in plant mutants. Multiple studies have shown that plants impaired in SA-signaling are highly susceptible to biotrophic and hemibiotrophic pathogens [103,104,105]. These findings contrast with those of a study published by Ameye et al. [44], which showed that pretreatment with Z-3-HAC inhibits SA responsive gene expression, while activating a JA-related defense response corresponds to the gene expression of wheat seedlings upon infection with the hemibiotroph *F. graminearum*. Although the expression analysis of the above-mentioned genes indicates that Z-3-HAC may act as a direct activator of SA-related defense response in potato plants against *P. infestans*, elucidating the detailed mechanisms involved in SA/JA-mediated defense pathways highlights a key challenge for conducting further research. Overall, the results of multiple studies together with the findings presented in the current work plainly demonstrate the role of GLVs in the activation of plant defense responses against pathogens.

## 5. Conclusions

The present study has shown that GLV Z-3-HAC has a strong inhibitory effect on *P. infestans* genotypes in planta, resulting in lower sporulation intensity compared to untreated plants. The tested GLV was able to reduce late blight severity around 70% in a very susceptible potato cultivar, ‘Bintje’, with no visible phytotoxic effects as the infection progressed. Similarly Z-3-HAC exposure conferred remarkable protection against *P. infestans* infection by prompting HR symptom development over time. Reduced disease severity in treated plants with Z-3-HAC was accompanied by higher expression levels of the *stRBOHb* and *stCDPK4* genes involved in ROS production, resulting in enhanced protection from subsequent infection of the hemibiotrophic *P. infestans*. Collectively, our current work may provide new insights into metabolic engineering strategies through the purposeful modification of GLV-derived compounds to enhance defense responses in potato plants, opening a potential avenue toward environmental technology for future work. Although more comprehensive studies addressing GLV-exposed plants are needed, widespread use of GLVs in management practices remains a challenge, largely associated with high-cost procedures.

## Figures and Tables

**Table 1:** Genes and their primer sequences for the analysis of gene expression using quantitative reverse transcription PCR (RT-qPCR).

Gene	Primer Sequence (5′-3′): Forward (F), Reverse (R)	Function	Reference
*stPR-1*	F: GGGAGAAGCCAAACTACAA R: TGAAATGAACCACCATCC	Marker gene in SA pathway	[51]
*stLOX*	F: CAGATCAGGCCCCGTTAATG R: CCTGTAAGTCCACCTTCACTTGTTG	Marker gene in JA synthesis	[52]
*stWRKY1*	F: GGTTCTTGGGACTAATGG R: GTTTTTGACAGCCTTTTG	Transcriptional factor in SA pathway	[51]
*EIN3*	F: CGACTCTGCTGCTACCGATGG R: GGTTCTTTCACTCTCAGGTTGCTC	Transcriptional factor in ET pathway	[53]
*stRBOHb*	F: GCTTCAATCAATCCAACAAGC R: GCGCCAATTTGGTCTAGC	Marker gene related to ROS production	This study
*stCDPK4*	F: ACCATTCTGGGCTGAAACAC R: CCGGATGAGATCTTTTGCAC	Transcriptional factor in SA pathway; marker gene related to ROS production	This study
*β-tubulin*	F: ATGTTCAGGCGCAAGGCTT R: TCTGCAACCGGGTCATTCAT	Reference gene	[53]
*actin*	F: CATCCTGTCCTCCTAACTGAAGCC R: TCACCAGAGTCCAACACAATACCG	Reference gene	[53]
